# Ribosomal protein uL3 targets E2F1 and Cyclin D1 in cancer cell response to nucleolar stress

**DOI:** 10.1038/s41598-019-51723-7

**Published:** 2019-10-28

**Authors:** Annalisa Pecoraro, Pietro Carotenuto, Giulia Russo, Annapina Russo

**Affiliations:** 1 Department of Pharmacy, University of Naples “Federico II”, Via Domenico Montesano 49, 80131 Naples, Italy; 2 The Institute of Cancer Research, Cancer Therapeutics Unit 15 Cotswold Road, Sutton, London SM2 5NG UK

**Keywords:** Epithelial-mesenchymal transition, Chemotherapy

## Abstract

Several experimental strategies in the treatment of cancer include drug alteration of cell cycle regulatory pathways as a useful strategy. Extra-ribosomal functions of human ribosomal protein L3 (uL3) may affect DNA repair, cell cycle arrest and apoptosis. In the present study, we demonstrated that uL3 is required for the activation of G1/S transition genes. Luciferase assays established that uL3 negatively regulates the activity of E2F1 promoter. Induced ribosome-free uL3 reduces Cyclin D1 mRNA and protein levels. Using protein/protein immunoprecipitation methods, we demonstrated that uL3 physically interacts with PARP-1 affecting E2F1 transcriptional activity. Our findings led to the identification of a new pathway mediated by uL3 involving E2F1 and Cyclin D1 in the regulation of cell cycle progression.

## Introduction

The cell cycle is a regulatory process by which cells grow, replicate their DNA and divide. It is controlled by a complex series of multiple, different signaling pathways. In cancer, as a consequence of genetic mutations, the strictly controlled process fails to correctly function resulting in uncontrolled cell proliferation^1^.

Perturbations of rRNA synthesis and/or processing are closely related to cell cycle alteration and cancer progression^2^. Ribosome biogenesis takes place in the nucleolus. Although the nucleolus represents mainly the site for ribosomal biogenesis and assembly, it is now clear that it is associated with many different cellular functions^3^. Recent data show that nucleolus acts as a sensor of cellular stress and responds by reorganizing its morphological architecture and protein content^4^. Nucleolar stress has been demonstrated to activate p53-dependent and p53-independent stress response signaling pathways with consequent cell cycle arrest and/or apoptosis^5^.

A group of ribosomal proteins (RP) affect cell cycle progression through the activation of different mechanisms. To cite, in Jurkat T-lymphoma cells the induced production of uL30 leads to cell cycle arrest in G1 phase; the overexpression of eL15 enhances cell proliferation; and the down-regulation of eL15 suppresses tumorigenicity of cancer cells in nude mice. RPs extraribosomal functions are also needed for normal cell proliferation. For example, uS3 is a microtubule-associated protein (MAP) and its depletion is associated to metaphase arrest and alteration in chromosome movement during cell division^6^.

We have recently demonstrated that the over-expression of uL3 causes cell cycle arrest in G1 phase by modulating p21 levels^7–10^. However, the mechanism through which, in presence of uL3, coordination between nucleolar stress and cell cycle progression occurs has yet to be elucidated.

Cell cycle progression through G1 phase is an activity strictly controlled in cells, it can be separated in early-G1 trascriptional activation of early genes, mid-G1 activation of cyclin D/cdk4/6, and late-G1 activation of cyclin E/cdk2^11^. In particular, the phosphorylation of retinoblastoma protein (pRb) family mediated by cdk/cyclin complexes is necessary for the transition from the G1 to the S phase of cell cycle. Phosphorylation of pRb leads to the release of E2F1 which in turn up-regulates the transcription of genes required to entry into late G1/S phase and further^12^. E2F1 function is subject to complex control mechanisms during cell cycle progression. In fact, prolonged activation of E2F1 expression is associated to G1 checkpoint activation and apoptosis, that prevent inappropriate reinitiation of DNA synthesis^13^. The comprehension of the molecular mechanisms controlling G1/S transition is crucial to identify new strategies to maintain a correct regulation of cell cycle.

In this study, we demonstrate that upon drug-induced nucleolar stress, uL3 accumulates in the nucleolus and localizes in the nucleoplasm. Consequently, uL3 affects E2F1 expression levels and the activity of E2F1 promoter; acts as negative regulator of cyclin D1 expression at mRNA and protein level; interacts with PARP-1 and influences PARP-1 mediated trans-activation of E2F1 promoter. Taken together, our data show that uL3 critically contributes to regulate cell cycle progression through the control of the expression and the activity of relevant genes involved in the G1/S transition of cell cycle.

## Results

### uL3 status influences cell migration and EMT program

We have previously demonstrated that uL3 status is associated to chemoresistance. In fact, we demonstrated that uL3ΔHCT 116^p53−/−^ cell line in which uL3 levels were strongly lower than in parental cell line^10^ resulted resistant to different anticancer drugs^14,15^. To further characterize the effects of uL3 silencing, we investigated the effect of low levels of uL3 on cell proliferation, cell motility and *epithelial–mesenchymal transition* (EMT), a migratory cellular program associated with tumor development and metastasis.

**Figure 1:**
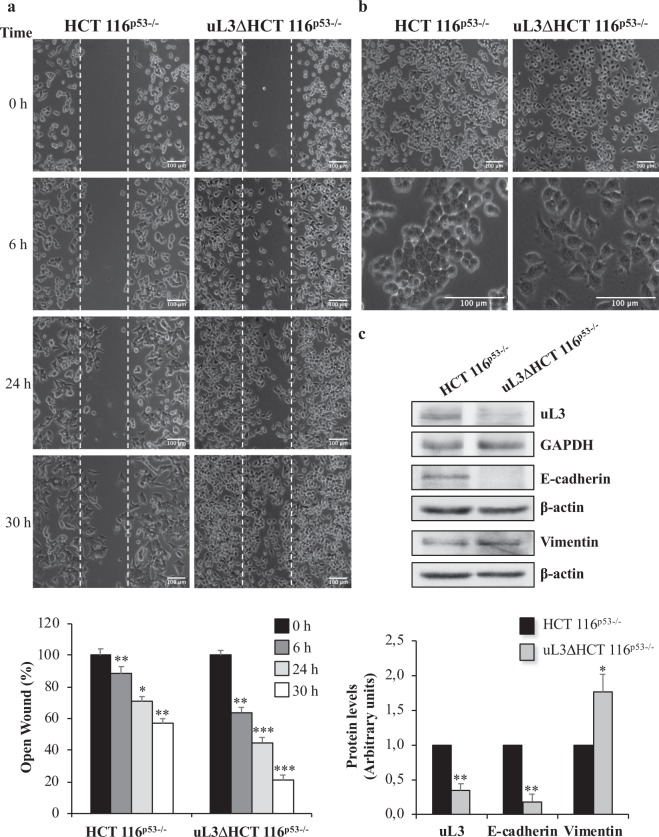
Effects of uL3 on cell migration and EMT program. (**a**) Wound widths in HCT 116^p53−/−^ and uL3∆HCT 116^p53−/−^ were measured at 0, 6, 24 and 30 h on 3 fields per well and averaged. Data are expressed as the fold-decrease of area respect to control (time 0) set as 100%. (**b**) Representative bright-field microscope images of HCT 116^p53−/−^ and uL3∆HCT 116^p53−/−^ cell lines. Scale bar: 100 µm. (**c**) Representative western blot analysis of uL3 and EMT markers. Protein extracts from HCT 116^p53−/−^ and uL3∆HCT 116^p53−/−^ cells were analyzed by western blotting with the indicated antibodies. GAPDH and β-actin were used as loading controls. Full-length blots are presented in Supplementary Fig. S7. Quantification of signals is shown. Bars represent the mean of triplicate experiments; error bars represent the standard deviation. *p < 0.05; **p < 0.01 vs. HCT 116^p53−/−^ cells set at 1.

Our results showed that uL3ΔHCT 116^p53−/−^ cells exhibited rates of proliferation comparable to parental cells (Supplementary Fig. S1). The wound healing ability of these cells was markedly increased in time dependent manner when compared to the wound healing ability observed in HCT 116^p53−/−^ cells (Fig. 1a, Supplementary Fig. S2). Quantitative analysis showed that after 30 h, HCT 116^p53−/−^ cells filled about 50% of the wound area while uL3ΔHCT 116^p53−/−^ cells filled about 80% of the wound area, demonstrating that uL3ΔHCT 116^p53−/−^ cells closed the wound faster than HCT 116^p53−/−^ cells. We also observed that the higher ability of uL3ΔHCT 116^p53−/−^ cells to migration was associated to morphological changes. More specifically, the low expression of uL3 in these cells was correlated to a characteristic EMT (Fig. 1b, Supplementary Fig. S3). In fact, analysis of the expression of EMT-related markers in uL3ΔHCT 116^p53−/−^ cells, measured by western blotting, showed a significant decrease of the epithelial marker E-cadherin and an increase of the mesenchymal marker vimentin (Fig. 1c).

All these results indicated that uL3ΔHCT 116^p53−/−^ cells, in which uL3 levels were reduced of about 70% compared to those in parental cell line, displayed rates of proliferation similar to the parental cell line, an increase in cell motility and a characteristic EMT phenotype.

### uL3 localizes in the nucleoplasm upon Act D exposure

The observed important role of uL3 in cell motility and de-differentiation, prompted us to explore extra-ribosomal functions of uL3 possibly leading to enhance cell responsiveness to anticancer treatments. Published data report that the alteration in wound healing ability and EMT transition correlates with changes in cell cycle regulators as cyclins, cdks and CKI (refs. ^16–18^). We have previously demonstrated that upon drug-induced nucleolar stress, uL3 as ribosome-free form can function mainly as transcriptional factor leading to cell cycle arrest and/or apoptosis^5^.

To approach the issue, primarily we monitored the intracellular localization of ribosome-free uL3 in condition of nucleolar stress. To this aim, HCT 116^p53−/−^ cells were transiently transfected with a plasmid expressing uL3 fused to GFP and treated for 18 h with low dose (5 nM) of Act D. Act D is a transcription inhibitor that blocks the RNA polymerase during the elongation step. High doses of Act D inhibit the transcription of all RNA species. At lower concentrations, i.e. 5 nM, Act D specifically inhibits RNA polymerase I driven transcription activating nucleolar stress^9,19^.

**Figure 2:**
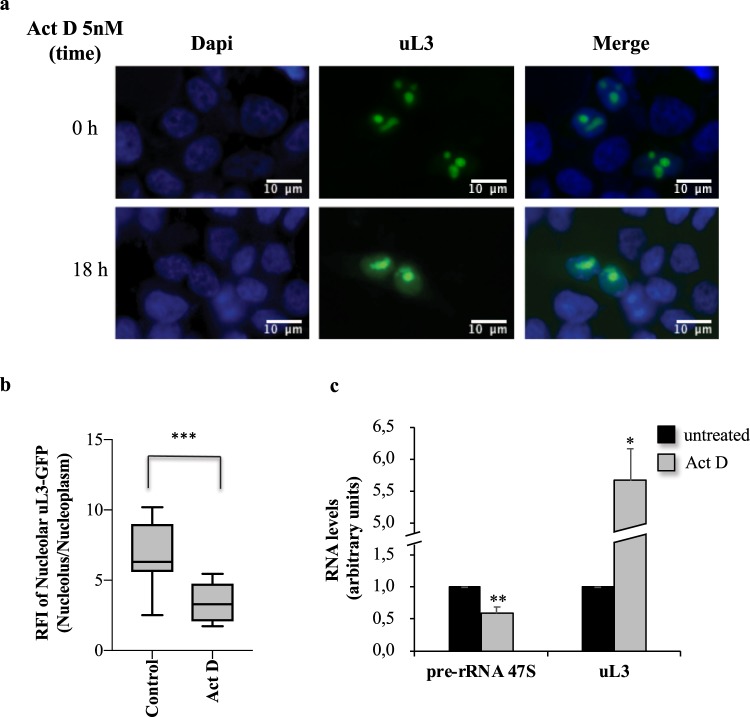
uL3 localizes in the nucleoplasm upon Act D exposure. (**a**) Representative fluorescent microscopy images of HCT 116^p53−/−^ cells transiently transfected with pGFP-uL3 and treated with Act D 5 nM for 18 h. DAPI is used as a nuclear stain and shown in blue; GFP-uL3 dependent fluorescence is shown in green. Scale bar: 10 μm. (**b**) Quantification of signal was shown. Nucleolar/nucleoplasmic RFI ratio of uL3-GFP (n = 31) were displayed. Mean ± s.e.m. Unpaired t-test. ***P < 0.001. (**c**) RT-qPCR of total RNA extracted from HCT 116^p53−/−^ cells treated with Act D 5 nM for 18 h with primers specific for uL3 and 47 S pre-rRNA (Table 1). Bars represent the mean of triplicate experiments; error bars represent the standard deviation. *p < 0.05; **p < 0.01 vs. untreated cells set at 1.

As shown in Fig. 2a and in Supplementary Fig. S4, in untreated cells uL3 protein distributed mainly in the nucleolus according to its role of ribosomal component. These data were also confirmed by experiments of biochemical fractionation demostrating that uL3 localizes in the nucleolus same as nucleolin, a well known marker of the nucleolus (Supplementary Fig. S5).

Upon Act D induced nucleolar stress, ribosome-free uL3 re-distributes and localizes largely also in the nucleoplasm (Fig. 2a,b). These results together with our previous findings demonstrating that in condition of nucleolar stress uL3 dissociates from the ribosome^9^, have led us to propose that in condition of Act D-induced nucleolar stress ribosome-free uL3, dissociates from the nucleolus and relocalizes in the nucleoplasm as shown in Fig. 2a.

Nevertheless, results of RT-qPCR analysis shown in Fig. 2c demonstrated that also the level of uL3 mRNA increased in this condition. Consequently, we cannot exclude that the localization of uL3 in the nucleoplasm may be due to an increase in the level of uL3 mRNA with consequent synthesis of uL3 protein that localizes, in condition of nucleolar stress, primarily in the nucleoplasm.

Reduced levels of pre-rRNA 47S confirmed the induction of nucleolar stress caused by Act D treatment (Fig. 2c).

### Role of uL3 on the expression of cell cycle and cell proliferation related genes

Nucleoplasm localization of uL3 upon nucleolar stress, alteration in cell motility and EMT transition prompted us to plan experiments to define its role in regulating cell cycle progression. Primarily, we analyzed the expression levels of cell cycle-related genes in condition of nucleolar stress, in presence or in absence of uL3.

**Figure 3:**
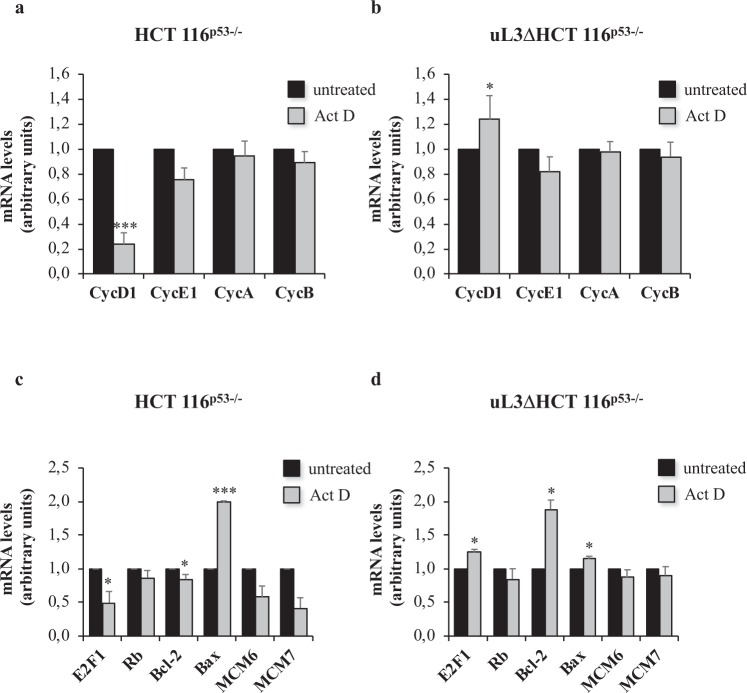
Act D treatment is associated to the uL3-mediated regulation of cell cycle and cell proliferation related genes. Total RNA from HCT 116^p53−/−^ cells (**a**,**c**) and uL3ΔHCT 116^p53−/−^ cells (**b**,**d**), untreated or treated with Act D 5 nM for 18 h, was subjected to RT-qPCR with primers specific for the indicated genes (Table 1). Quantification of signals is shown. Bars represent the mean of triplicate experiments; error bars represent the standard deviation. *p < 0.05; ***p < 0.001 vs. untreated cells set at 1.

HCT 116^p53−/−^ and uL3ΔHCT 116^p53−/−^ cells were treated with Act D 5 nM for 18 h. Then, total RNA extracted from cell lysates was analyzed by RT-qPCR with specific primers for the expression of proteins regulating cell cycle progression as Cyclin D1, Cyclin E1, Cyclin A and Cyclin B (Table 1). As shown in Fig. 3a, analysis of RNA levels of cell cycle checkpoint proteins in HCT 116^p53−/−^demonstrated that upon nucleolar stress a strong decrease of Cyclin D1 was observed associated to a lower decrease also in Cyclin E1. The expression of Cyclin A and Cyclin B was not significantly altered suggesting that these regulatory proteins were not affected by drug treatment. Previous data demonstrated that the reduction of Cyclin D1 expression was associated to an increased sensitivity of cells to drug-induced apoptosis^20^, consequently we analyzed the expression of anti-apoptotic protein Bax and pro-apoptotic Bcl-2. As expected, the increase of ribosome free uL3 caused by drug treatment was associated to a strong induction of Bax and a decrease of Bcl-2 in HCT 116^p53−/−^ cells (Fig. 3c). In addition to cyclins, transition from G1 to the S phase of the cell cycle requires the inactivation of the retinoblastoma protein (Rb) family, cell cycle inhibitors at the key G1/S restriction point, and the consequent activation of E2F1^11,21^. This transcription factor regulates the expression of various proteins including Cyclin D1 and MCM2–7 that form the preinitiation complex for DNA replication^11,13^. In particular, it has been demonstrated that free E2F1 may contribute to either the activation or to the inhibition of the expression of Cyclin D1 depending on the cells context^22^ and as a consequence free E2F1 can induce either apoptosis than proliferation^22^. Our results demonstrated that in HCT 116^p53−/−^ cells, Act D induced nucleolar stress did not affect Rb amount but caused a significant reduction of E2F1 levels which was associated to a decrease of Mcm6 and Mcm7 levels, thus indicating a reduction of DNA synthesis (Fig. 3c). In order to understand whether the observed effects were dependent on uL3 status, we analyzed the expression profiles of tested genes when uL3 gene expression was silenced. To this aim, uL3ΔHCT 116^p53−/−^ cells were treated with Act D for 18 h and total RNA extracted was analyzed with the same primers (Table 1). Of interest, when uL3 was silenced, the treatment with Act D resulted in an increase of Cyclin D1 and E2F1 expression levels (Fig. 3b,d). As in parental cell line, the expression of other members of cyclin family was not altered (Fig. 3b). Furthermore, Act D was uneffective on MCM6 and MCM7 expression (Fig. 3d). Of interest, the treatment with Act D in absence of uL3 failed to induce apoptosis as demonstrated by Bcl-2 and Bax protein levels.

**Table 1:** Oligonucleotide sequences used in qPCR analysis.

Gene	Sequence
CycA	Forward: 5′–TTCATTTAGCACTCTACACAGTCACGG–3′ Reverse: 5′–TTGAGGTAGGTCTGGTGAAGGTCC–3′
CycB	Forward: 5′–CAGTCAGACCAAAATACCTACTGGGT–3′ Reverse: 5′–ACACCAACCAGCTGCAGCATCTTCTT–3′
CycD1	Forward: 5′– ACGGCCGAGAAGCTGTGCATC–3′ Reverse: 5′–CCTCCGCCTCTGGCATTTTGGAG–3′
CycE1	Forward: 5′–TGAGCCGAGCGGTAGCTGGT–3′ Reverse: 5′–GGGCTGGGGCTGCTGCTTAG–3′
Bax	Forward: 5′–CCCGAGAGGTCTTTTCCGAG–3′ Reverse: 5′–CCAGCCCATGATGGTTCTGAT–3′
β-actin	Forward: 5′–CCAACCGCGAGAAGATGA–3′ Reverse: 5′–CCAGAGGCGTACAGGGATAG–3′
Bcl-2	Forward: 5′–ATGTGTGTGGAGAGCGTCAACC–3′ Reverse: 5′–GCATCCCAGCCTCCGTTATC–3′
E2F1	Forward: 5′–GTGTAGGACGGTGAGAGCAC–3′ Reverse: 5′–TCAAGGGTAGAGGGAGTTGG–3′
MCM6	Forward: 5′–ATCCCTCTTGCCAAGGATTT–3′ Reverse: 5′–GAAAAGTTCCGCTCACAAGC–3′
MCM7	Forward: 5′–CACGGAGTCTCTCAGCACAG–3′ Reverse: 5′–AACATCTGTCTGATGGGGGA–3′
Rb	Forward: 5′–ATTCTGCATTGGTGCTAAAAG–3′ Reverse: 5′–CTCCTGTTCTGACCTCGC–3′
uL3	Forward: 5′–CAAAGGCTACAAAGGGGT–3′ Reverse: 5′–CTCAGTGCGGTGATGGTAG–3′
pre-rRNA 47S	Forward: 5′–GCTGACACGCTGTCCTCTG–3′ Reverse: 5′–ACGCGCGAGAGAACAGCAG–3′

These data suggest Cyclin D1 and E2F1 are important players in uL3 cell response to nucleolar stress induced by Act D in colon cancer cells harboring p53 deletion.

### uL3 negatively regulates Cyclin D1 mRNA and protein stability

Since our data demonstrated that uL3 status influences Cyclin D1 levels in colon cancer cells, we proceeded in the attempt to characterize the specific molecular targets of uL3 along the expression process of Cyclin D1.

**Figure 4:**
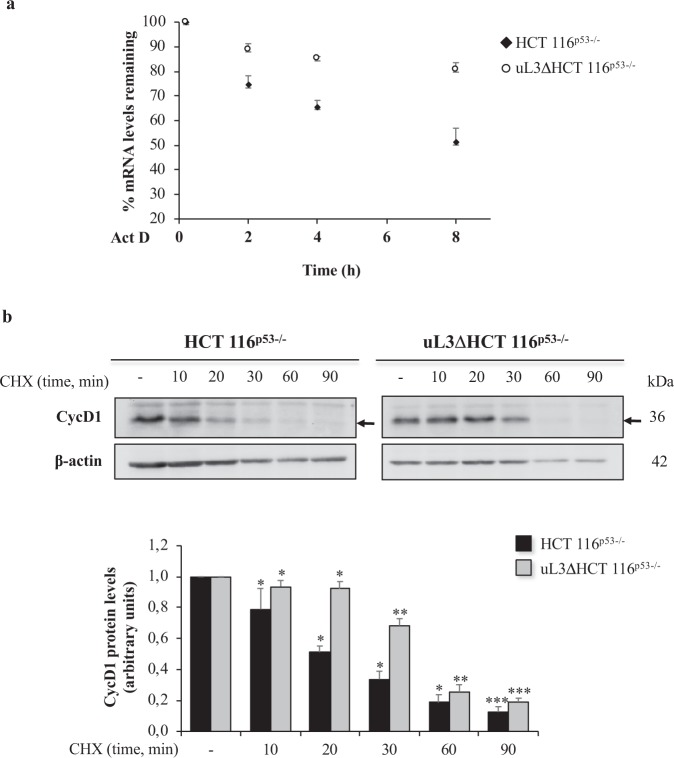
Effects of uL3 on Cyclin D1 mRNA and protein stability. (**a**) HCT 116^p53−/−^ and uL3ΔHCT 116^p53−/−^ cells were treated with Act D (2 μg/mL) for 8 h. At the indicated time points (0, 2, 4 and 8 h), total RNA was isolated and the mRNA levels of Cyclin D1 and β-actin were determined by RT-qPCR. The relative amount of Cyclin D1 mRNA in untreated cells was set to 100% and the percentage of Cyclin D1 mRNA in cells treated with Act D was calculated accordingly. (**b**) HCT 116^p53−/−^ and uL3ΔHCT 116^p53−/−^ cells were treated with CHX (100 μg/mL) for 10, 20, 30, 60 and 90 min. Then, cell lysates were immunoblotted with anti-Cyclin D1 and anti-β-actin antibodies. Full-length blots are presented in Supplementary Fig. S8. Quantification of signals is shown. Bars represent the mean of triplicate experiments; error bars represent the standard deviation. ***P < 0.001, **P < 0.01, *P < 0.05 vs. untreated cells set at 1.

A number of studies have shown that cellular accumulation of Cyclin D1 is under tight control and its expression is regulated at multiple levels, including mRNA and protein stability^23^. Consequently, we investigated whether uL3 could affect Cyclin D1 expression at mRNA and protein levels. To this aim, cells were incubated with Act D (2 μg/mL) to inhibit nascent mRNA synthesis. Total RNA was obtained from HCT 116^p53−/−^ and uL3ΔHCT 116^p53−/−^ cells at the indicated times (2, 4 and 8 h) and Cyclin D1 mRNA levels were analyzed by qRT-PCR. The results showed that in HCT 116^p53−/−^ the amount of Cyclin D1 mRNA was lower than in uL3ΔHCT 116^p53−/−^ at all time points. In particular, the levels of Cyclin D1 mRNA in HCT 116^p53−/−^ were approximately 50% lower compared to Cyclin D1 mRNA in uL3ΔHCT 116^p53−/−^ after 8 h of Act D treatment (Fig. 4a). These results indicate that the down-regulation of Cyclin D1 mRNA levels observed in HCT 116^p53−/−^ following Act D treatment could be due partially to uL3-mediated decrease in Cyclin D1 mRNA stability. Next, we performed experiments to test a role of uL3 in the control of Cyclin D1 protein stability. To this aim HCT 116^p53−/−^ and uL3ΔHCT 116^p53−/−^ cells were treated with cycloheximide at different time points (10, 20, 30, 60 and 90 min). Then, cell lysates were immunoblotted with anti-Cyclin D1 and anti-β-actin antibodies. Analysis of the rate of disappearance of the protein following cycloheximide treatment clearly demonstrated that silencing of uL3 was associated to an increase of Cyclin D1 stability (Fig. 4b).

All these data suggest that uL3 is involved in the regulation of Cyclin D1 expression by altering the stability either at mRNA than at protein levels.

### Role of uL3 on E2F1 protein levels and transcription activity of E2F1 gene

**Figure 5:**
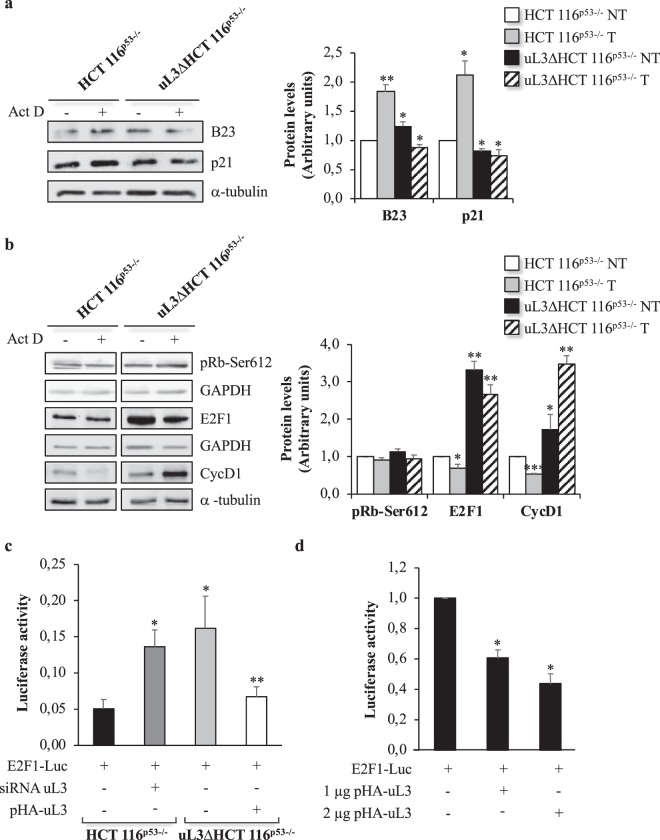
Effects of uL3 on cell cycle related gene expression and E2F1 promoter activity. (**a**) Representative western blotting of B23 and p21, (**b**) pRb-Ser612, E2F-1 and CycD1. HCT 116^p53−/−^ and uL3ΔHCT 116^p53−/−^ cells were treated with Act D 5 nM for 18 h. After the treatment, protein extracts from the samples were analyzed by western blotting with antibodies against indicated proteins. α-tubulin and GAPDH were used as loading controls. Full-length blots are presented in Supplementary Figs S9 and S10. Quantification of signals is shown. Bars represent the mean of triplicate experiments; error bars represent the standard deviation. **P < 0.01, *P < 0.05 vs. untreated HCT 116^p53−/−^ cells set at 1. (**c**) HCT 116^p53−/−^ were transiently co-transfected with E2F1 promoter-driven reporter construct pGL2-AN (E2F1-Luc) and uL3 siRNA, uL3ΔHCT 116^p53−/−^ cells were transiently co-transfected with E2F1-Luc and pHA-uL3. Luciferase activities were measured after 24 h. Data are presented after normalizing transfection efficiency using the Renilla luciferase reporter gene. Bars represent the mean of triplicate experiments; error bars represent the standard deviation. **P < 0.01, *P < 0.05 vs. E2F1-Luc transfected cells. (**d**) HCT 116^p53−/−^ cells were transiently transfected with E2F1-Luc alone or with 1 μg or 2 μg of pHA-uL3. Luciferase activities were measured after 24 h. Data are presented after normalizing transfection efficiency using the Renilla luciferase reporter gene. Bars represent the mean of triplicate experiments; error bars represent the standard deviation.*P < 0.05 vs. E2F1-Luc transfected cells set at 1.

It is known that in late G1 hyperphosphorylation of Rb by cyclin-dependent kinases (cdks) results in the release of E2F1. Free E2F1 transactivates the expression of E2F1-responsive genes important for induction of S phase progression^11,24^. To understand the biological effect of the observed reduction of Cyclin D1 expression in presence of uL3, we firstly analyzed the protein levels of E2F1 and Rb in condition of ActD induced nucleolar stress in presence or absence of uL3. To this aim, HCT 116^p53−/−^ cells and uL3ΔHCT 116^p53−/−^ cells were treated with Act D 5 nM and 18 h later, proteins from the samples were extracted and analyzed by western blotting. As shown in Fig. 5a, in HCT 116^p53−/−^ cells, the treatment with Act D caused an increase of B23 and p21 levels, markers of nucleolar stress. In this condition, the intracellular amounts of pRb-Ser612 did not change while the E2F-1 and Cyclin D1 levels decreased (Fig. 5b). These results indicated that, in condition of nucleolar stress, in cells expressing uL3, the amounts of E2F1 and Cyclin D1 decreased significatively respect to the levels of unstressed cells. In untreated uL3ΔHCT 116^p53−/−^ cells, p21 and B23, as expected, were down-regulated (Fig. 5a). Of interest, E2F1 and Cyclin D1 resulted up-regulated in these cells, while the levels of pRb-Ser612 were not notably altered (Fig. 5b). These data suggest that the lack of uL3 in uL3ΔHCT 116^p53−/−^ cells activates a new uL3/E2F-1/CycD1 pathway that is independent from Rb status. It is important to note that drug treatment in absence of uL3 produced only a slight decrease of E2F1 whereas Cyclin D1 expression was up-regulated extensively in this condition (Fig. 5b).

The effect of uL3 on the expression profile of E2F1 prompted us to investigate the ability of uL3 to control the activity of E2F1 promoter by using a reporter luciferase assay. To this aim, HCT 116^p53−/−^ and uL3ΔHCT 116^p53−/−^ cells were transiently transfected with a plasmid carrying E2F1 full length promoter and, 24 h later, luciferase activity in cell lysates was analyzed. We found that the activation of E2F1 promoter was dramatically increased in absence of uL3 (Fig. 5c).

To verify that uL3ΔHCT 116^p53−/−^ cells did not acquire additional alterations in addition to uL3 depletion, we have analyzed the role of uL3 in the transactivation of E2F1 promoter in condition of transient uL3 silencing. To this aim, HCT 116^p53−/−^ cells were transiently transfected with siRNA specific for uL3 (Supplementary Fig. 6a). 48 h later, cell lysates were subjected to luciferase assay. Results shown in Fig. 5c demonstrate that transient depletion of uL3 caused an effect similar to that obtained with stable silencing of uL3 indicating that the alteration of E2F1 promoter activity was specifically due to the absence of uL3. In addition, HCT 116^p53−/−^ cells were transiently transfected with pHA-uL3, a plasmid expressing uL3 fused to HA (Supplementary Fig. 6b). 24 h later, luciferase activity was measured. As shown in Fig. 5c, the effect observed upon uL3 silencing was rescue by uL3 transfection.

These results clearly indicated that uL3 plays a critical role in the regulation of E2F1 transcriptional activity. In particular, results of luciferase reporter analysis suggest that uL3 acts as a specific repressor of E2F1 promoter activity.

To further verify this hypothesis, we performed luciferase assay in HCT 116^p53−/−^ cells in condition of uL3 overexpression. Cells were transiently transfected with increasing amount of pHA-uL3. 24 h later, luciferase activity in cell lysates was measured. As shown in Fig. 5d, the enforced expression of uL3 was associated to the down-regulation of E2F1 gene promoter trans-activation in a dose-dependent manner.

### PARP-1 is a novel uL3-interacting protein

**Figure 6:**
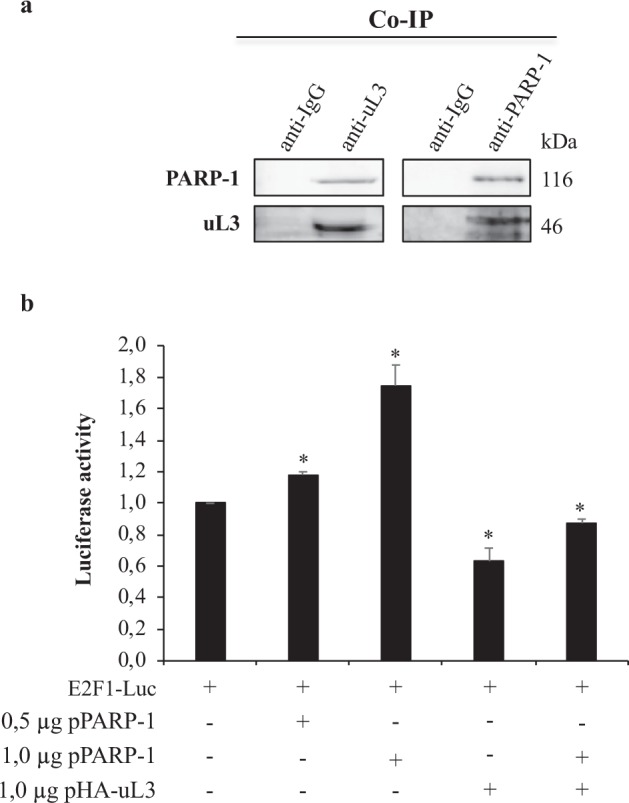
uL3 binds PARP-1 and regulates PARP-1 mediated E2F1 promoter activation. (**a**) *In vivo* binding of uL3 and PARP-1. uL3 and PARP-1 were specifically immunoprecipitated from HCT 116^p53−/−^ cell extracts with antibodies against the endogenous uL3 and PARP-1. Immunoprecipitates were separated by SDS–PAGE and immunoblotted with antibodies versus PARP-1 and uL3 respectively. Note the absence of signal in IgG immunocomplex. Full-length blots are presented in Supplementary Fig. S11. (**b**) HCT 116^p53−/−^ cells were transiently transfected with E2F1 promoter-driven reporter construct pGL2-AN (E2F1-Luc) alone or co-transfected with 0.5 or 1 μg of pPARP-1 plasmid or 1 μg of pHA-uL3 alone or in combination with 1 μg of pPARP-1 plasmid. Luciferase activities were measured after 24 h. Data are presented after normalizing transfection efficiency using the Renilla luciferase reporter gene. Bars represent the mean of triplicate experiments; error bars represent the standard deviation. *P < 0.05 vs. E2F1-Luc transfected cells set at 1.

To better understand the role of uL3 on the transactivation of E2F1 promoter we wondered whether uL3 physically interacted with Poly(ADP-ribose) polymerase 1 (PARP-1), the main E2F1 positive regulator in cells^25^. To address this issue, we conducted co-immunoprecipitation experiments. Figure 6a shows the results of the experiments in which uL3 and PARP-1 were specifically immunoprecipitated from cell extracts by using antibodies against the endogenous proteins. Immunoprecipitated proteins were separated by SDS–PAGE and the presence of uL3 and PARP-1 was investigated by western blotting in the reciprocally immunoprecipitated complexes. The results of these experiments showed that uL3 and PARP-1 were co-immunoprecipitated, thus indicating that they can associate *in vivo*. Note the absence of signal in IgG immunocomplex.

Next, we investigated the functional relevance of the protein complex uL3-PARP-1 in the control of E2F1 promoter activation. To this aim, reporter luciferase assays were performed. Specifically, the E2F1 reporter plasmid was transiently transfected in HCT 116^p53−/−^ cells together with increasing amounts of expression plasmids coding for uL3 and/or PARP-1. 24 h after transfection, cells were analyzed for luciferase activity. As expected, the promoter activity was up-regulated upon PARP-1 over-expression. In contrast, the enforced expression of uL3 negatively regulates E2F1 promoter. Of note, in condition of uL3 over-expression, PARP-1 fails to overcome the uL3 mediated down -regulation of E2F1 promoter (Fig. 6b). These data suggest that uL3 controls PARP-1 mediated activation of E2F1 promoter.

## Discussion

Some ribosomal proteins have extra-ribosomal functions not strictly related to protein synthesis and it is now clear that a connection between alterations in ribosomal biology and cancer exists^26^. In particular, an association between alteration in the expression of ribosomal proteins and cancer development and/or chemioresistance is often observed. Loss of 60 S uL18 and uL16 proteins has been seen in multiple myeloma and acute myeloid leukemia^27^; eL19, eL8 and eL37 have been found over-expressed in prostate cancer^28,29^; eL15 and eL19 in gastric cancer^6^. All these findings raise the question on how alterated levels of ribosomal proteins increase malignancy. uL3 is a crucial component of the ribosomal peptidyltransferase center and has a role in aminoacyl-tRNA binding, peptidyltransferase activity, translational frame maintenance and elongation^30^. uL3 is a member of a subset of RPs that as free proteins are implicated in various important events of the cell life. As free form regulates its own expression through the generation of mRNA isoforms that are target of nonsense-mediated mRNA decay^31,32^. We have previously demonstrated that uL3 is also a key determinant in cellular stress response to common chemoterapeutic drugs as 5-FU, OHP and Act D in *p53*-mutated lung and colon cancer cells^8,9^. Restoration of the uL3 protein level re-sensitize the resistant cells to drugs by regulating the levels of p21 and CBS proteins^14,15^. Published evidences from our group indicates that Act D is able to trigger nucleolar stress modulating uL3 levels and in turn p21 activity with consequent cell cycle arrest or apoptosis^9^. Here, we show that in condition of Act D induced nucleolar stress, ribosome free uL3 translocates from the nucleolus to the nucleoplasm where it can exert extra-ribosomal functions (Fig. 2a). To better characterize this alternative role, we performed experiments in colon cancer cells expressing uL3 and in a sub-line stably depleted of uL3 (uL3ΔHCT 116^p53−/−^ cells). We show that when uL3 expression was switched off an increase in cell migration was observed as demonstrated by the enhanced wound healing ability of these cells compared to that observed in HCT 116^p53−/−^ cells (Fig. 1a). Starting from this observation and the concept that EMT is a tissue remodeling process reactivated during wound healing and in cancer^33^ we analyzed the expression profile of E-cadherin and vimentin proteins that are considered two key markers associated with EMT^33^.

In this study, we found that in uL3ΔHCT 116^p53−/−^ cells the low expression of uL3 is associated to the EMT transition (Fig. 1b,c), which results in a more aggressive, invasive cancer phenotype. These data indicate a role for uL3 loss in cancer as an inducer of EMT. Accordingly, pharmacological inhibition of Pol I, which causes the accumulation of uL3 ribosome-free form and activates its extra-ribosome functions^5^, lowered the presence of pro-invasive mesenchymal proteins and reduced cellular invasiveness^34^.

Our previous data demonstrated that uL3 positively targets p21 to induce cell cycle arrest and/or apoptosis. Consistent with the known function of p21 in inhibition of EMT^35^, it is plausible that the observed EMT upon depletion of uL3 was due to consequent lower levels of p21.

Acquisition of a migratory phenotype and EMT are hallmarks of human carcinoma cells that may be interconnected by molecular pathways controlling cell cycle progression^36^. By RT-qPCR experiments we demonstrated that in cells expressing uL3, Act D caused the down-regulation of E2F1 gene expression which was associated to a decreased expression of Cyclin D1 and the up-regulation of pro-apoptotic gene Bax (Fig. 3a,c). It has been demonstrated that E2F1 plays a central role in the control of the expression of Cyclin D1. The human Cyclin D1 promoter contains an E2F1 consensus site and E2F1 can repress Cyclin D1 gene expression^37^. Our data strongly suggest that uL3 is able to regulate E2F1 amount which in turn controls the expression of Cyclin D1.

In addition, it has been shown that E2F1 can induce apoptosis through a number of p53- independent mechanisms mainly due to E2F-mediated regulation of various pro-apoptotic genes^38^. According to this, the observed downregulation of E2F1 expression during nucleolar stress, required for apoptosis^39^, associated to a strong increase of the pro-apoptotic protein Bax (Fig. 3c).

Interestingly, in cells in which uL3 expression was switched off the treatment with Act D showed different effects; in fact, it was associated to the up-regulation of Cyclin D1, E2F1 and Bcl-2 gene (Figs 3b,d and 5b). These data clearly indicate that uL3 is essential to mediate cell cycle arrest induced by Act D^9^ by specifically regulating the expression of cell cycle (E2F1 and Cyclin D1) and cell proliferation (Bax and Bcl-2) genes.

Important to note that the phosphorylation status of Rb was not affected by Act D treatment in both cell lines (Fig. 5) indicating that the regulatory mechanisms mediated by uL3 of E2F1 activity differ from those generally reported^11,21^.

Hyperactivation of E2F1 observed in absence of uL3, has been shown to frequently contribute to malignant progression^40^. In accordance with these results, the lack of uL3 was associated to a strong increase of Cyclin D1 which correlates with a marked increase in cell motility and EMT transition observed in these cells. Cyclin D1, in fact, apart from its role in cell cycle dependent phosphorylation of Rb protein, plays a central role in the transcriptional regulation of genes involved in cell migration independently from its ability to interact with CDKs. More specifically, Cyclin D1 participates in RhoA-ROCK pathway by binding directly to p27 with consequent inhibition of the pathway. Inhibition of the Rho/ROCK2 pathway leads to a decrease in number of focal adhesion as well as actin stress fibers that results in increased cell motility^41^. The observed up-regulation of Cyclin D1 represents also a mechanism of chemoresistance. These data are in line with previous findings demonstrating that the over-expression of Cyclin D1 results in drug resistance in different cancers^20^. In particular, it has been shown that elevated Cyclin D1 expression in conditions of p53 deficiency promotes chemoresistance in Mantle Cell Lymphoma^42^. In addition, numerous evidences have demonstrated that the attenuation of Cyclin D1 expression resulted in enhanced chemosensitivity to anticancer drugs^20^. The expression of Cyclin D1 is highly regulated at different levels^23^. Here, we report a key role of uL3 in the control of Cyclin D1 intracellular amounts. In cells silenced of uL3, the stability of Cyclin D1 mRNA and protein was increased (Fig. 4a,b) and the levels of this protein were higher than in parental cell line (Fig. 5b). Furthermore, in HCT 116^p53−/−^ cells the up-regulation of uL3, after treatment with Act D, resulted in a reduction of about 50% in the Cyclin D1 protein levels (Fig. 5b). uL3 could regulate Cyclin D1 expression acting on the stability of mRNA and protein or indirectly by inhibiting E2F1 promoter transactivation (Fig. 5c, d). The uL3 mediated transcriptional regulation of E2F1 results in a reduction of E2F1 protein that is known positively regulated Cyclin D1 promoter activity^22^.

With the aim to examine the molecular mechanisms involved in the uL3 mediated down-regulation of E2F1 transcription we identified PARP-1, a nuclear enzyme essential for genomic stability and chromatin remodeling, as a new molecular target of uL3. Specifically, we demonstrated that uL3 and PARP-1 co-immunoprecipitate indicating that these proteins associate *in vivo* (Fig. 6a).

PARP-1 represents the main positive regulator of E2F1 in cells. Specifically, PARP-1 acts as a positive cofactor of E2F-1-mediated transcription. PARP-1 does not directly bind the E2F1 promoter but it interacts directly with E2F1 protein and increases the binding affinity of E2F1 to its consensus sequence^25^.

In addition, (ADP-ribosyl)ation status of E2F1 influences its ability to bind specific protein partners. To date, PARP-1 (ADP-ribosyl)ates E2F1 and in this way stabilizes the binding between E2F1 and other proteins as BIN1 tumor suppressor. When PARP-1 is deficient, hypo-poly(ADP-ribosyl)ated E2F1 releases the proapoptotic BIN1 and cells result more sensible to apoptosis^43^. To evaluate the role of uL3 in PARP-1 mediated activation of E2F1, we investigated the effects of PARP-1 on E2F1-mediated functions in the presence and absence of uL3.

Data from luciferase assay upon uL3 and PARP-1 co-expression show that uL3 acts as repressor of E2F1 promoter activity and suggest that uL3 could bind the positive regulator PARP-1 and sequester it from the E2F1 promoter (Fig. 6b).

**Figure 7:**
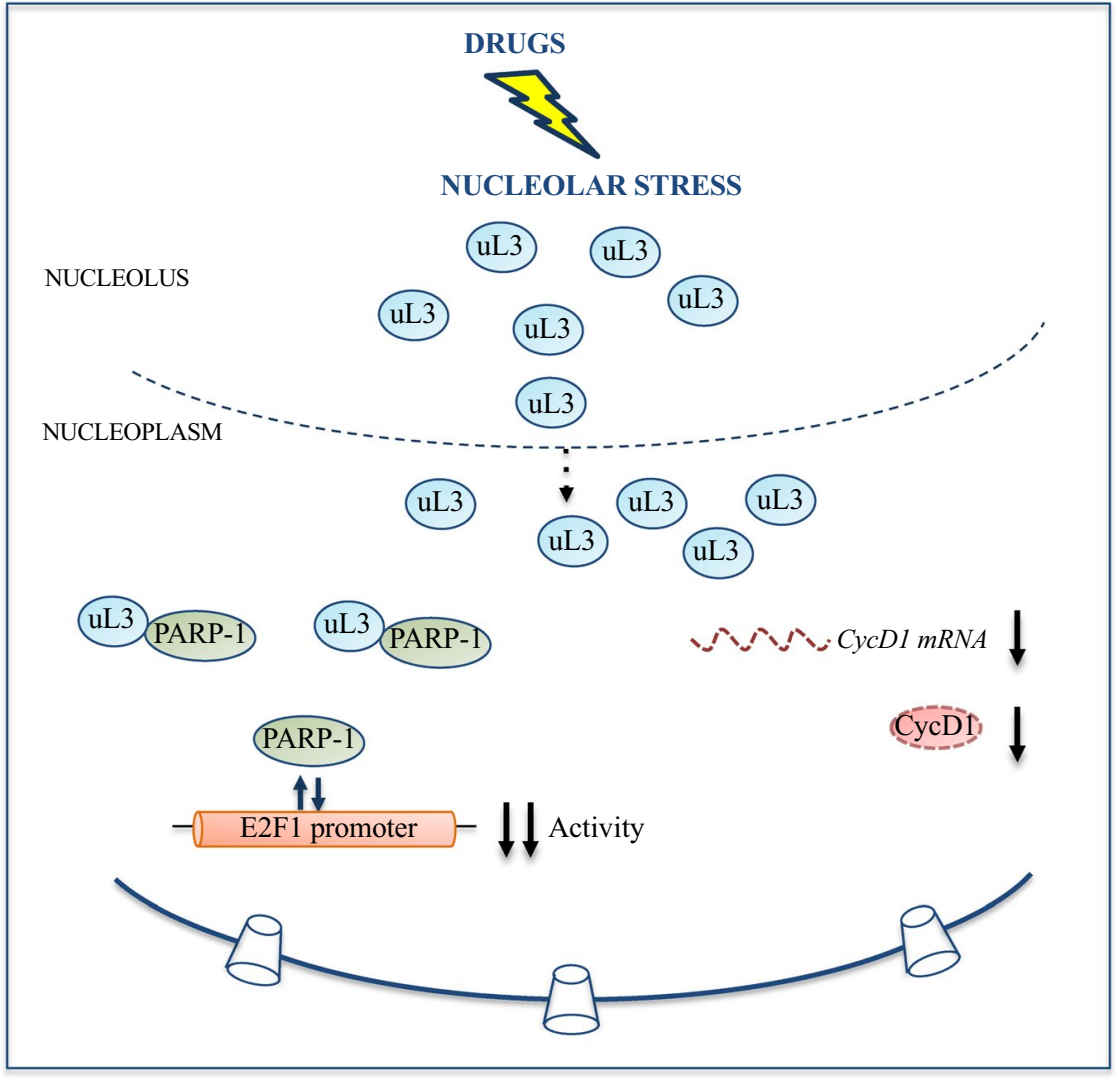
Schematic representation of proposed model. Upon drug induced nucleolar stress, ribosome free uL3 accumulates in the nucleolus and translocates into the nucleoplasm. Here, uL3 acts as a negative regulator of E2F1 promoter activity. In addition, uL3 inhibits Cyclin D1 protein half-life. These effects contribute to uL3 mediated cell response to nucleolar stress.

Taken together these results led us to propose a model in which upon drug induced nucleolar stress, ribosome free uL3 accumulates in the nucleolus and translocates into the nucleoplasm. Here, uL3 becomes a repressor of E2F1 promoter activity. uL3 interacts with PARP-1 and it could prevent its binding to the E2F1 promoter inhibiting in this way its transactivation. In addition, uL3 negatively affects Cyclin D1 expression at mRNA and protein levels. The uL3 mediated inhibition of E2F1 transactivation could be responsible at least in part of the lower levels of Cyclin D1 observed in condition of Act D induced nucleolar stress (Fig. 7).

In conclusion, our data unveil E2F1 and Cyclin D1 inactivation as a novel uL3 mediated cell response to nucleolar stress-based anticancer treatments.

## Methods

### Cell cultures, DNA, transfections and drug treatments

HCT 116^p53−/−^ cells and uL3ΔHCT 116^p53−/−^ a cell line derived from HCT 116^p53−/−^ cells and stably silenced for uL3^10^, were cultured in Dulbecco’s Modified Eagle’s Medium (DMEM) supplemented with 10% fetal bovine serum (FBS), 2 mM L-glutamine, penicillin-streptomycin 50 U/ml.

Drug treatments were performed by adding to cells Act D (Sigma). Transfection of pHA-uL3 plasmid, pGFP-uL3 plasmid, PARP-1 plasmid (Addgene, cod. 111575) and pGL2-AN containing the entire E2F-1 promoter (Addgene, cod. 20950) was performed in cells using Lipofectamin 2000 as previously described^44^. siRNA transfections were performed using Oligofectamine Reagent (Invitrogen) according to the manufacturer’s instructions.

### Wound healing assay

Cell motility was assessed using a wound healing assay as previously reported^8^.

### Fluorescence microscopy

HCT 116^p53−/−^ cells were plated on coverslips at a density of 2 × 10^4^ cells per well into 35 mm tissue culture plates and treated with 5 nM Act D. 18 h later, cells were analyzed as previously described^14,45^.

### Image processing and fluorescence signal quantifications

Fluorescence images obtained from immunofluorescence were processed in ImageJ software (version 1.49 v with 64 bit Java Platform; NIH) and exported as TIFF mode files in red/green/blue (RGB) channels. To normalize individual cell differences, in general 30 cells were examined for quantification of average fluorescent intensity. The absolute fluorescence intensity and line plots were obtained from bit channel files before the images were transferred to RGB channel files. The relative fluorescence intensity (RFI) was calculated as RFI = Ncl/Npl where Ncl was the absolute intensity in the nucleolus area, Npl was the average the average intensity in nucleoplasmatic region.

### RT-qPCR

Total RNA was isolated from cells as previously described^46^. RNA was first retrotranscribed using SensiFAST^TM^ cDNA Synthesis kit (Bioline) and then realtime PCR was carried out using SensiFAST SYBER® No-ROX kit. The primers are indicated in Table 1. The comparative Ct method was used to calculate the relative abundance of the mRNA and compared with that of β-actin expression^47^.

### Nucleolar isolation

HCT 116^p53−/−^ cells plated in 100 mm petri dishes were washed twice with 20 mL cold solution I (0,5 M sucrose, 3 mM MgCl_2_, 1X EDTA-free Roche protease inhibitor cocktail).

Collected cells were sonicated on ice at 50% power, 10 s on, 10 s off, for five cycles. The sonicated cell suspension was layered over 1,4 mL precooled solution II (1 M sucrose, 3 mM MgCl_2_, 1X EDTA-free Roche protease inhibitor cocktail) and centrifuged at 1800 × g for 10 min at 4 °C. The resulting pellet contained the isolated nucleoli. The samples were resuspended in 200 μl of 8 M urea and incubated at 56 °C for 40 min. Then, each sample was analyzed by western blotting.

### Immunoprecipitation and western blotting

Immunoprecipitation assay was performed as previously reported^48^. Briefly, whole cell lysate (1 mg) was incubated with 30 μl of protein A/G agarose beads coated with 5 μg of anti-uL3 (Primm, Milan, Italy) at 4 °C for 12 h. The beads were washed and boiled in the SDS sample buffer. The eluted proteins were loaded on 12% SDS-PAGE and detected by western blotting.

Western blotting analysis was performed as previously reported^49^. The membranes were challenged with anti-E2F1 (Elabscience), anti-uL3 (Primm, Milan, Italy), anti-PARP-1, anti-GAPDH (Cell signaling), anti-B23, anti-p21, anti-α-tubulin, anti-β-actin, anti-nucleolin, anti-vinculin, anti-HA (Santa Cruz Biotechnology). Proteins were visualized with enhanced chemiluminescence detection reagent according to the manufacturer’s instructions (Elabscience®).

### Luciferase assays

Luciferase assays were performed as previously reported^8^.

### Protein half-life analysis

Protein half-life analysis was performed as previously reported^50^. Briefly, cells were treated with Cycloheximide (CHX, Sigma-Aldrich, St. Louis, MO, USA) 0,1 mg/ml for different times, and subsequently harvested and lysed using RIPA lysis buffer (50 mM Tris-HCl pH 7.4, 1% NP40, 0,5% Na-deoxycolate, 150 mM NaCl, 1 mM Na3VO4, 1 mM NaF, 1X EDTA-free Roche protease inhibitor cocktail). Protein extracts from samples were analyzed by western blotting.

### Statistical analysis

Statistical analysis was performed as previously reported^51^. For image quantification, GraphPad Prism software (version 8.0) was used to analyse and plot all data. Statistical analysis was performed with two-tailed unpaired *t*-test with 95% confidence interval. In box and whisker plots, the box showed the top and bottom quartiles (25–75%) with a line at the median and the whiskers showed the minimum to the maximum values of all data. *P*-values indicated the significances of differences.

## Supplementary information

Supplementary Information
