# Supplementary material for: Ribosomal protein uL3 targets E2F1 and Cyclin D1 in cancer cell response to nucleolar stress
Source: Sci Rep. 2019 Oct 28;9:15431. doi: 10.1038/s41598-019-51723-7 (PMC6817900; doi:10.1038/s41598-019-51723-7)
Supplement: Supplementary file 1 — Supplementary Information [file 41598_2019_51723_MOESM1_ESM.pdf]

Ribosomal protein uL3 targets E2F1 and Cyclin D1 in cancer cell response to nucleolar stress

Annalisa Pecoraro, Pietro Carotenuto, Giulia Russo, Annapina Russo

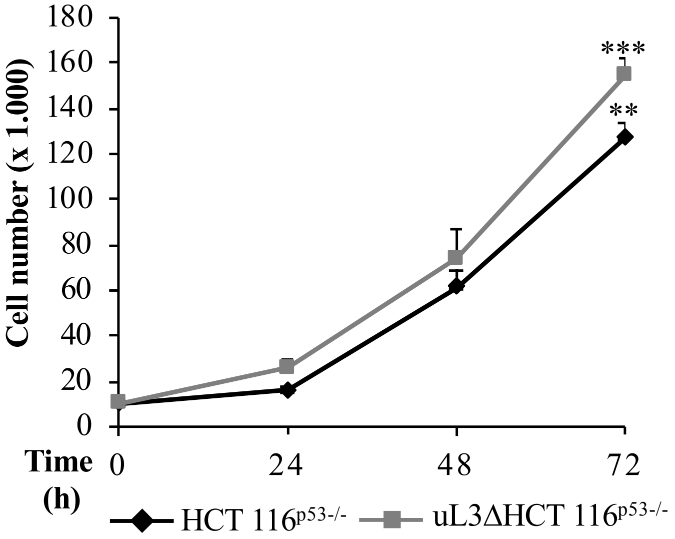

Supplementary Fig S1. Cell proliferation profile of uL3 deleted cell line. Proliferation rates in HCT 116$^{p53-/-}$ and uL3ΔHCT 116$^{p53-/-}$ cells were measured after seeding 10.000 cells and counted at 24, 48, 72 h using a hemocytometer. Data represent the mean of triplicate experiments; error bars represent the standard deviation. $^{**}p < 0.01$; $^{***}p < 0.001$.

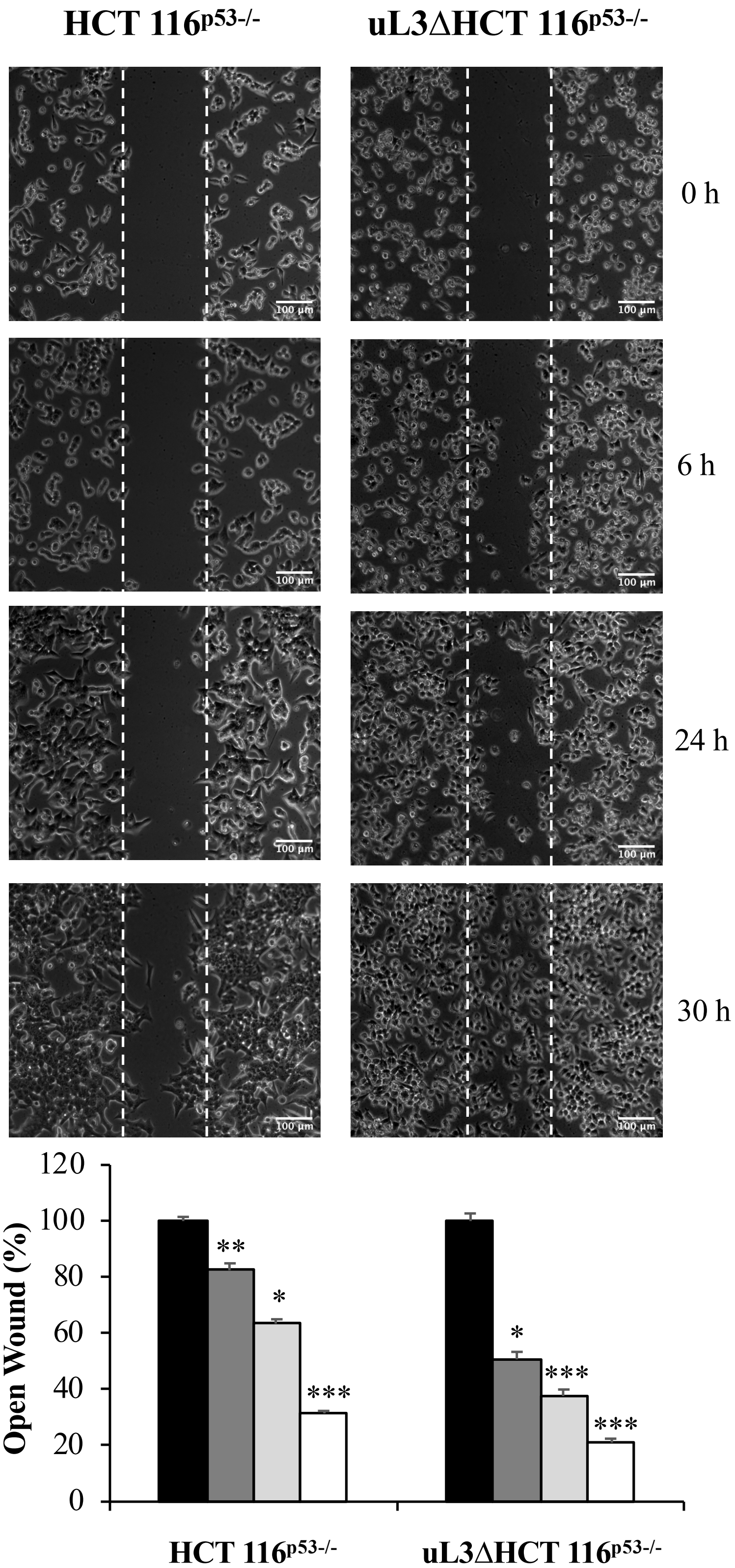

Supplementary Fig S2. Role of uL3 on cell migration. Wound widths were measured at 0, 6, 24 and 30 h on 3 fields per well and averaged. Data are expressed as the fold-decrease of area respect to control (time 0) set as 100%. Error bars represent the standard deviation. *$p < 0.05$; **$p < 0.01$; ***$p < 0.001$.

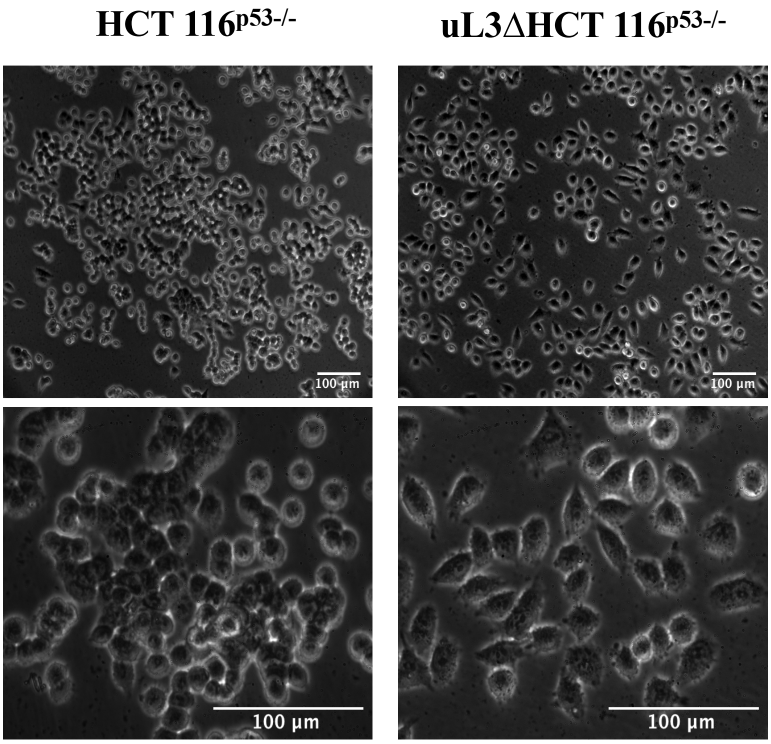

Supplementary Fig S3. Representative bright-field microscope images of HCT 116$^{p53-/-}$ and uL3ΔHCT 116$^{p53-/-}$ cell lines. Scale bar: 100 μm.

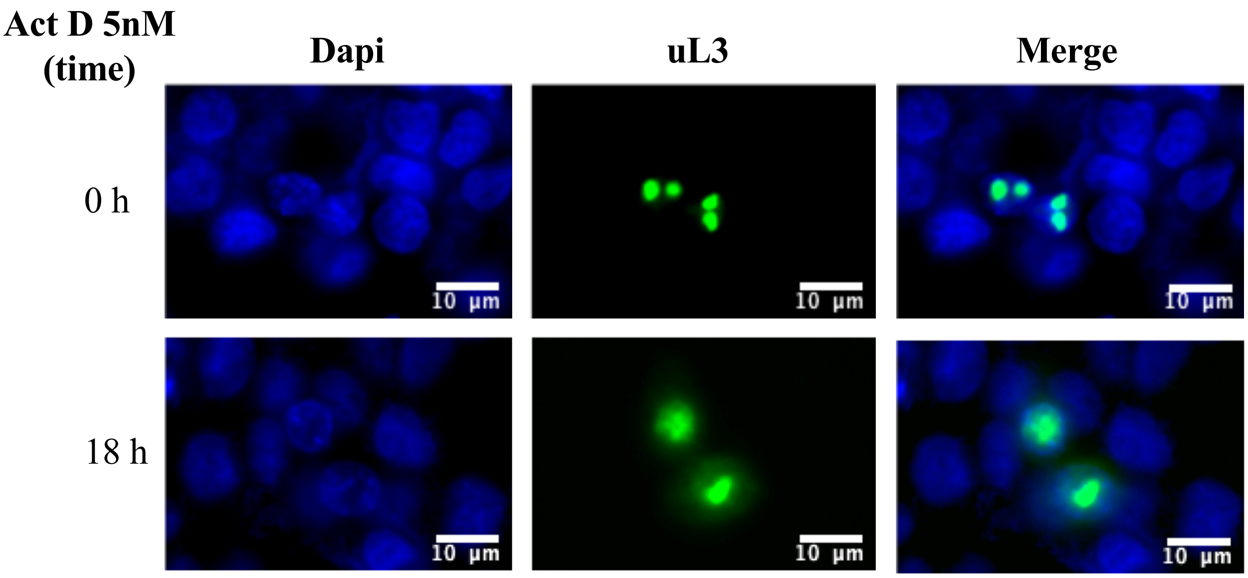

Supplementary Fig S4. uL3 localizes in the nucleus upon Act D exposure. Representative fluorescent microscopy images of HCT 116$^{p53-/-}$ cells transiently transfected with pGFP-uL3 and treated with Act D 5nM for 18 h. DAPI, used as nuclear stain, is shown in blue; GFP-uL3 dependent fluorescence is shown in green. Scale bar: 10 μm.

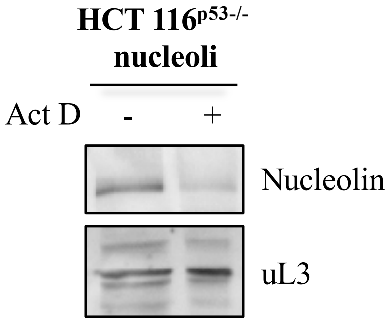

Supplementary Fig S5. HCT 116$^{p53-/-}$ cells were treated with 5nM Act D. 18 h later, cells were subjected to fractionation to isolate the nucleoli. Protein extracts from the samples were analyzed by western blotting with antibodies against uL3 and Nucleolin as marker of nucleolar fraction. Full-length blots are presented in Supplementary Fig. S12.

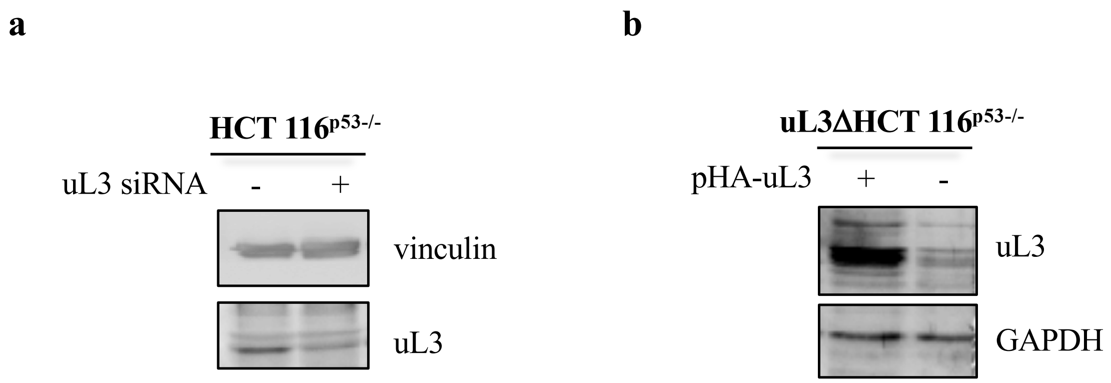

Supplementary Fig S6. (**a**) HCT 116$^{p53-/-}$ cells were transiently transfected with siRNA specific for uL3. 24 h later, cells were lysated and protein extracts from the samples were analyzed by western blotting with antibodies against uL3 and vinculin as loading control. (**b**) uL3ΔHCT 116$^{p53-/-}$ cells were transiently transfected with 1 μg of pHA-uL3. 24 h later, cells were lysated and protein extracts from the samples were analyzed by western blotting with antibodies against uL3 and GAPDH as loading control. Full-length blots are presented in Supplementary Fig. S13.

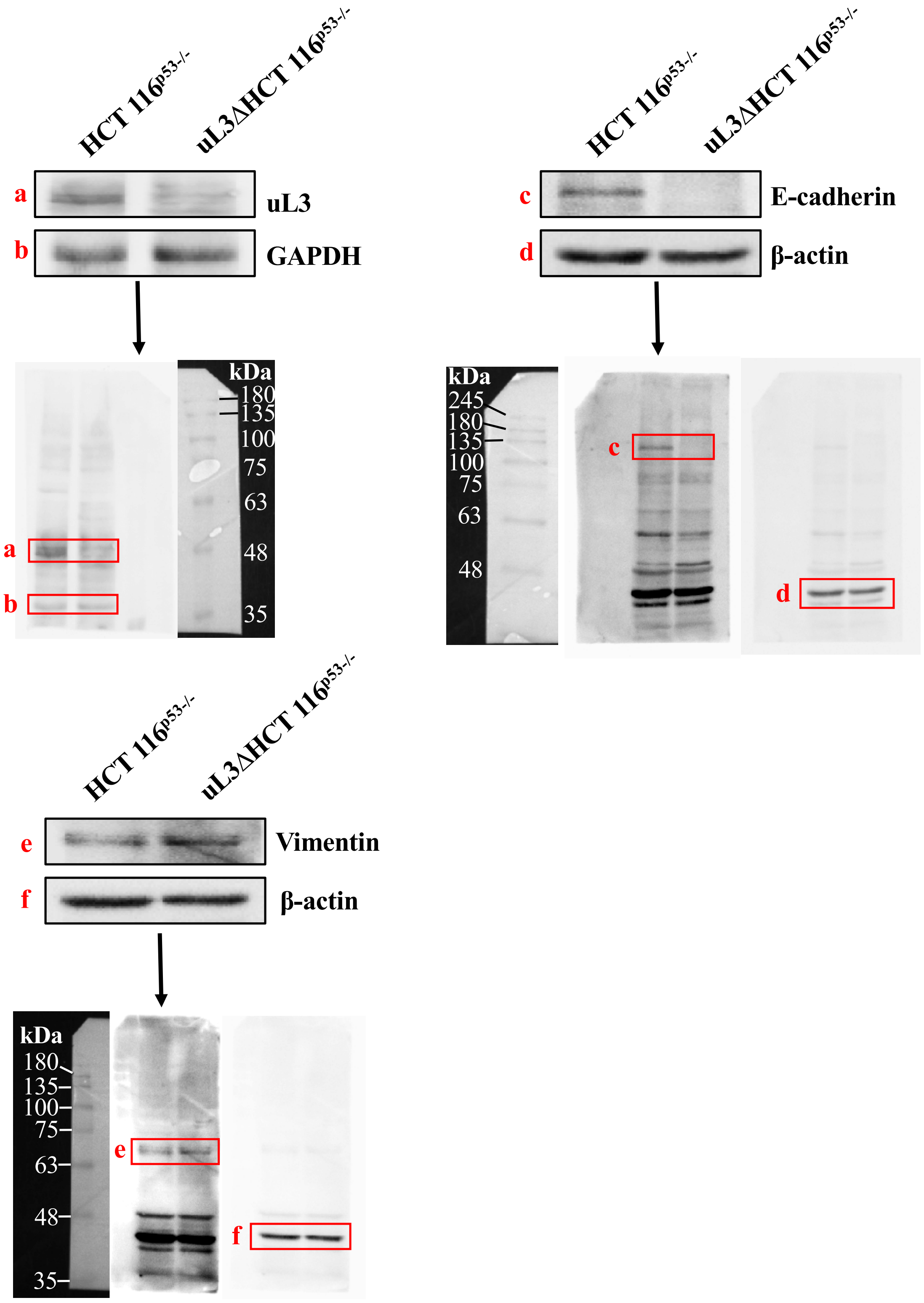

Supplementary Fig S7: full length blots of Fig 1c.

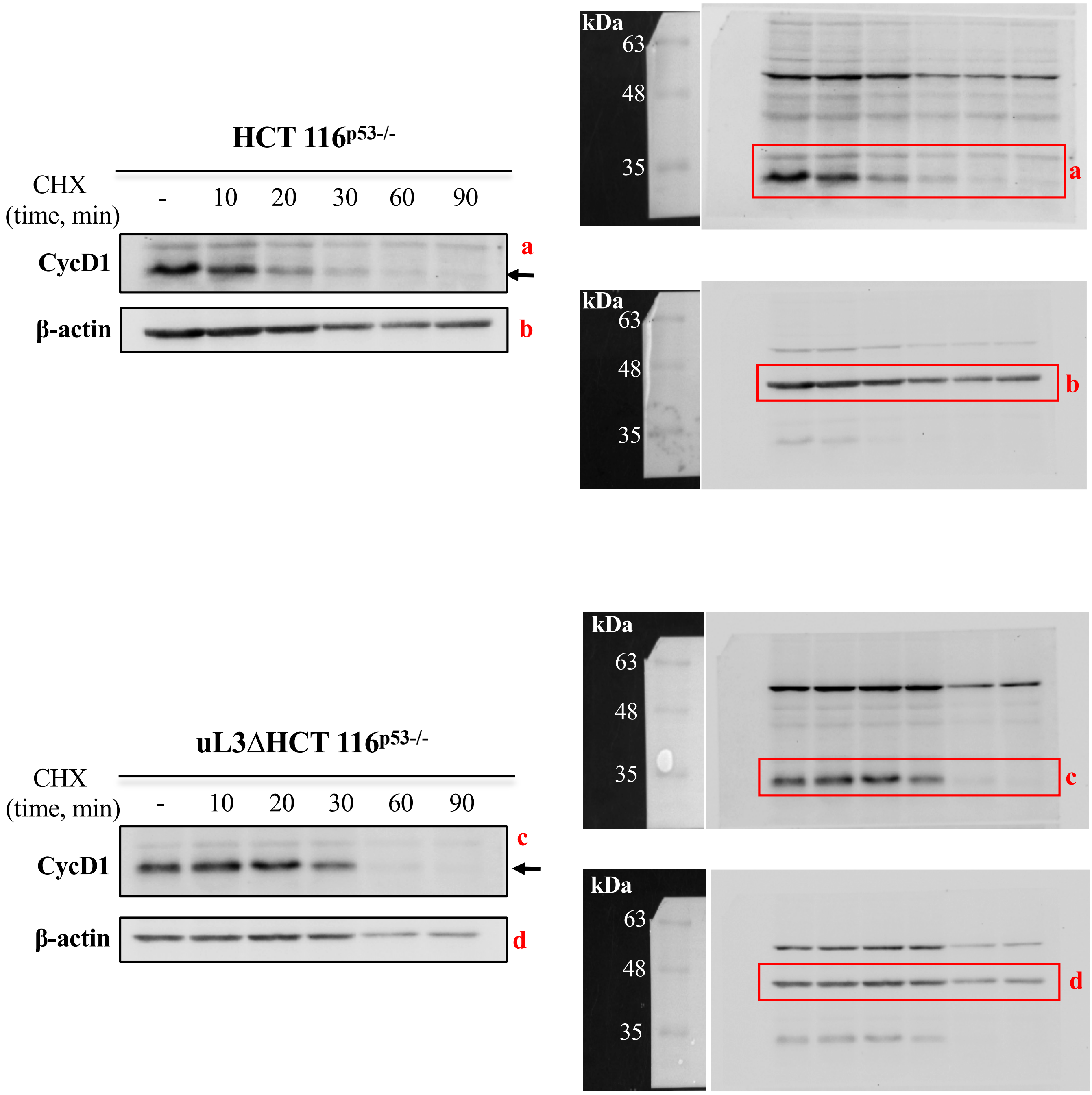

Supplementary Fig S8: full length blots of Fig 4b.

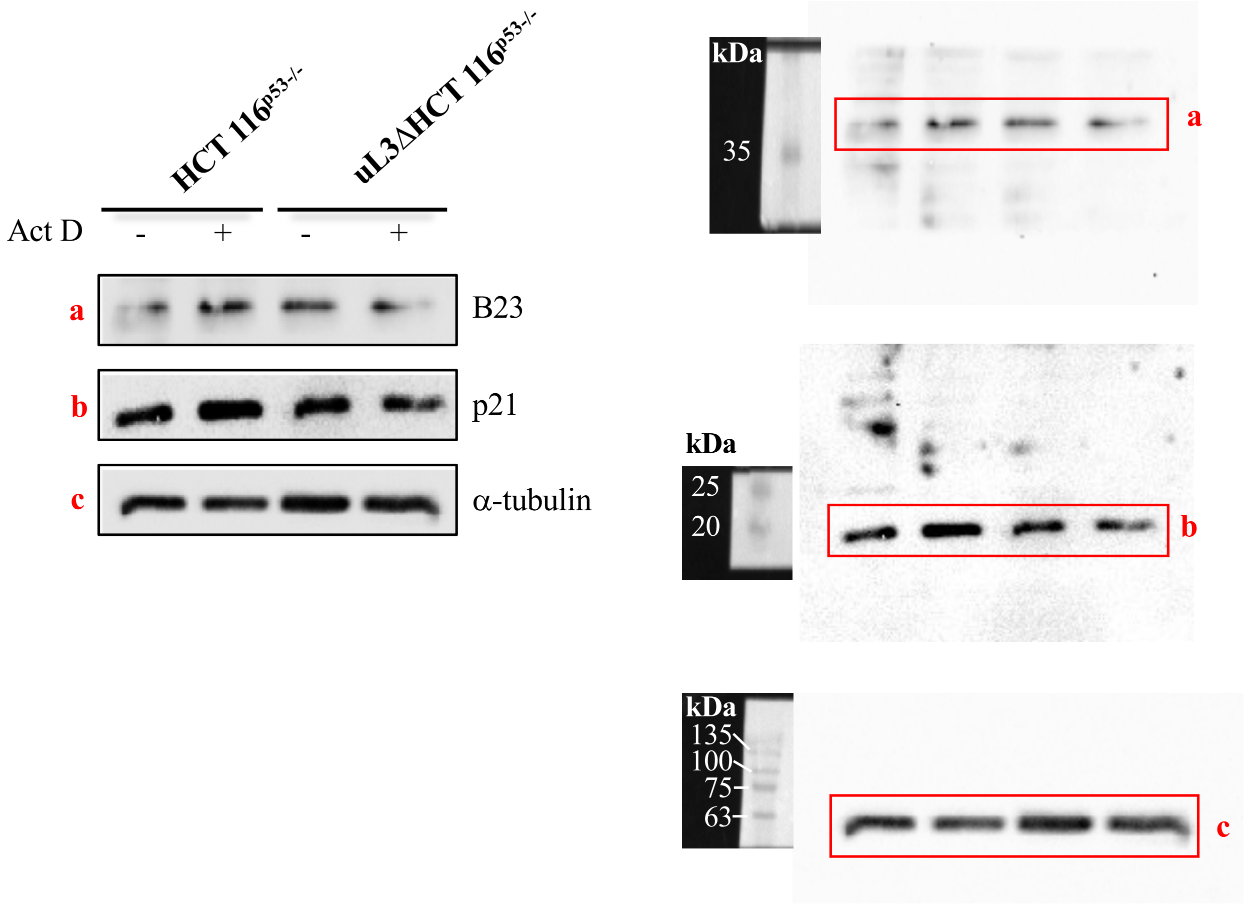

Supplementary Fig S9: full length blots of Fig 5a.

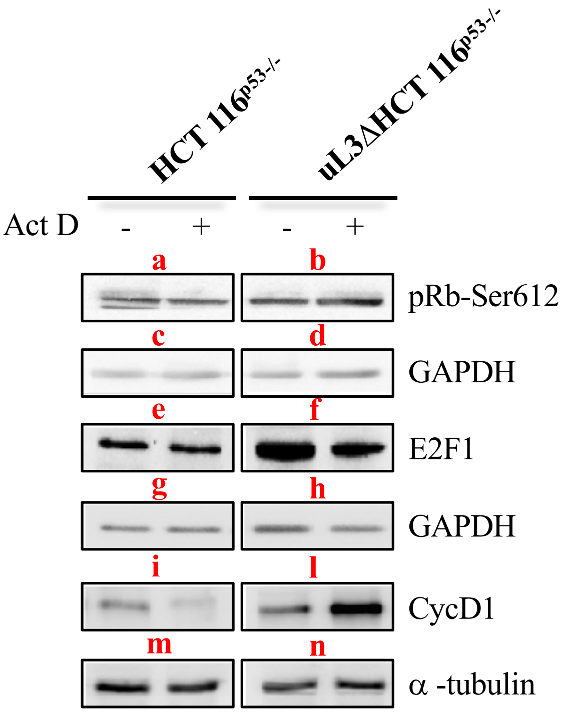

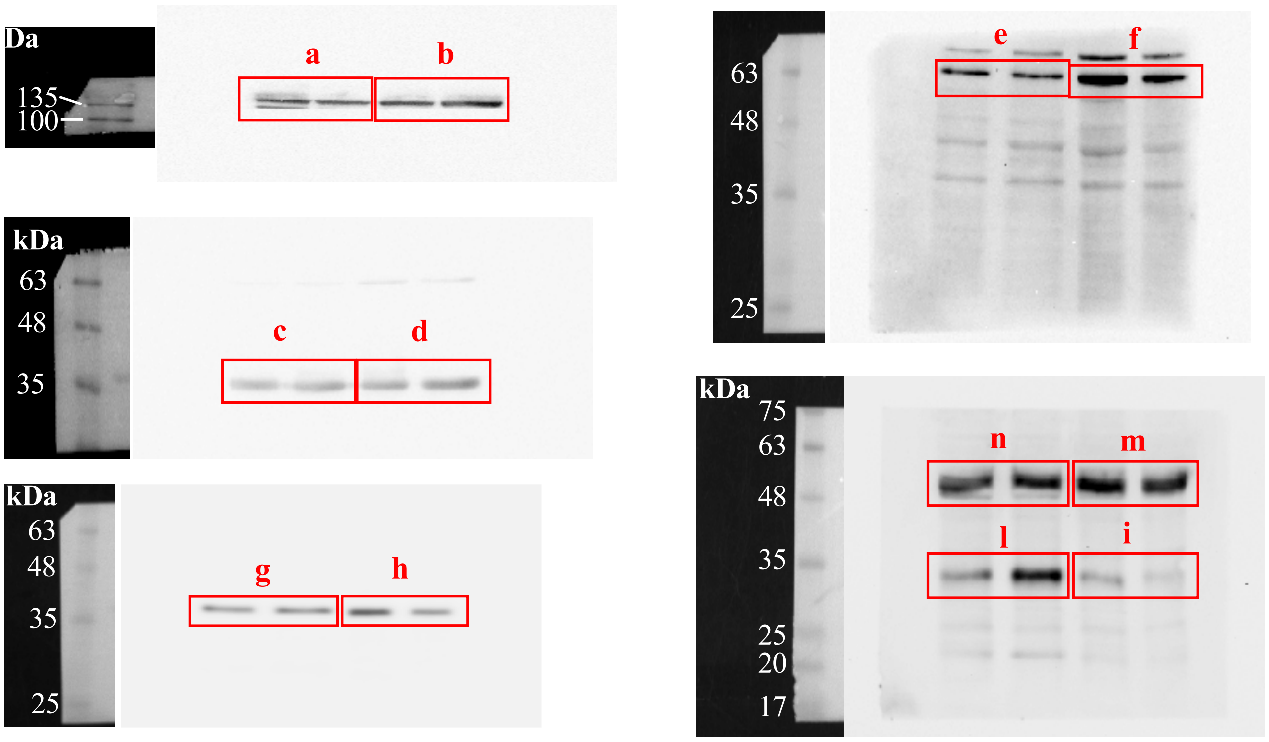

Supplementary Fig S10: full length blots of Fig 5b.

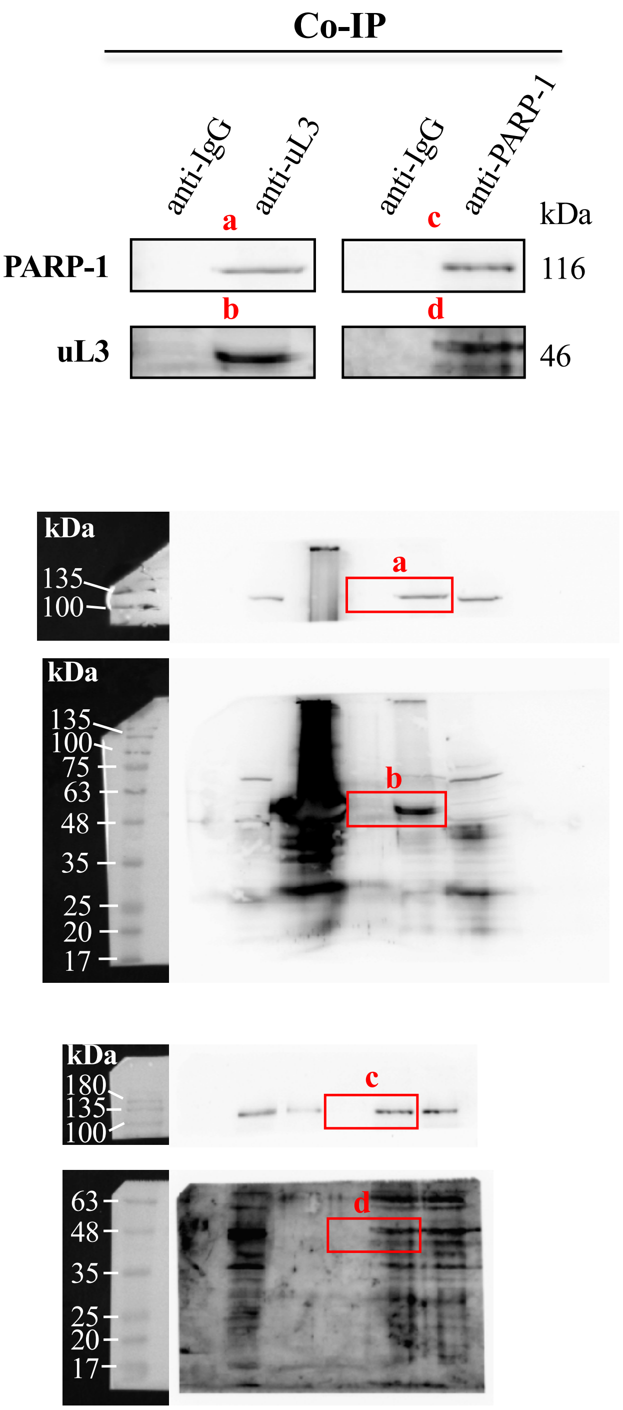

Supplementary Fig S11: full length blots of Fig 6a.

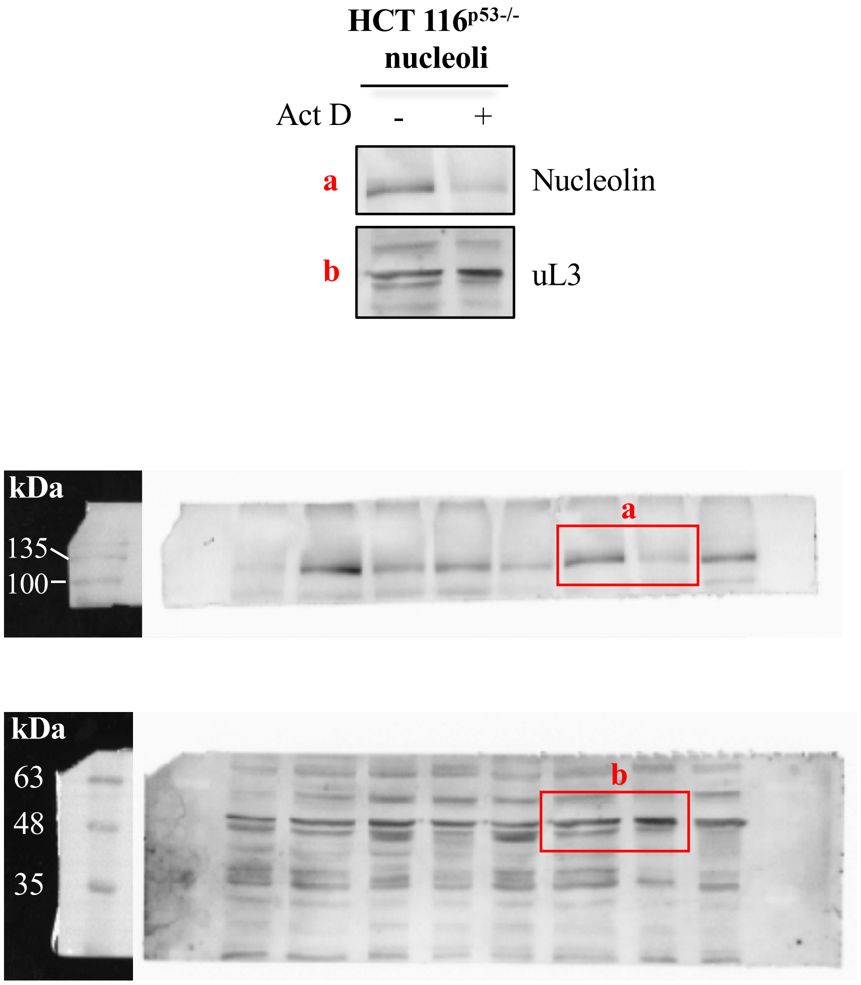

Supplementary Fig S12: full length blots of Fig S5.

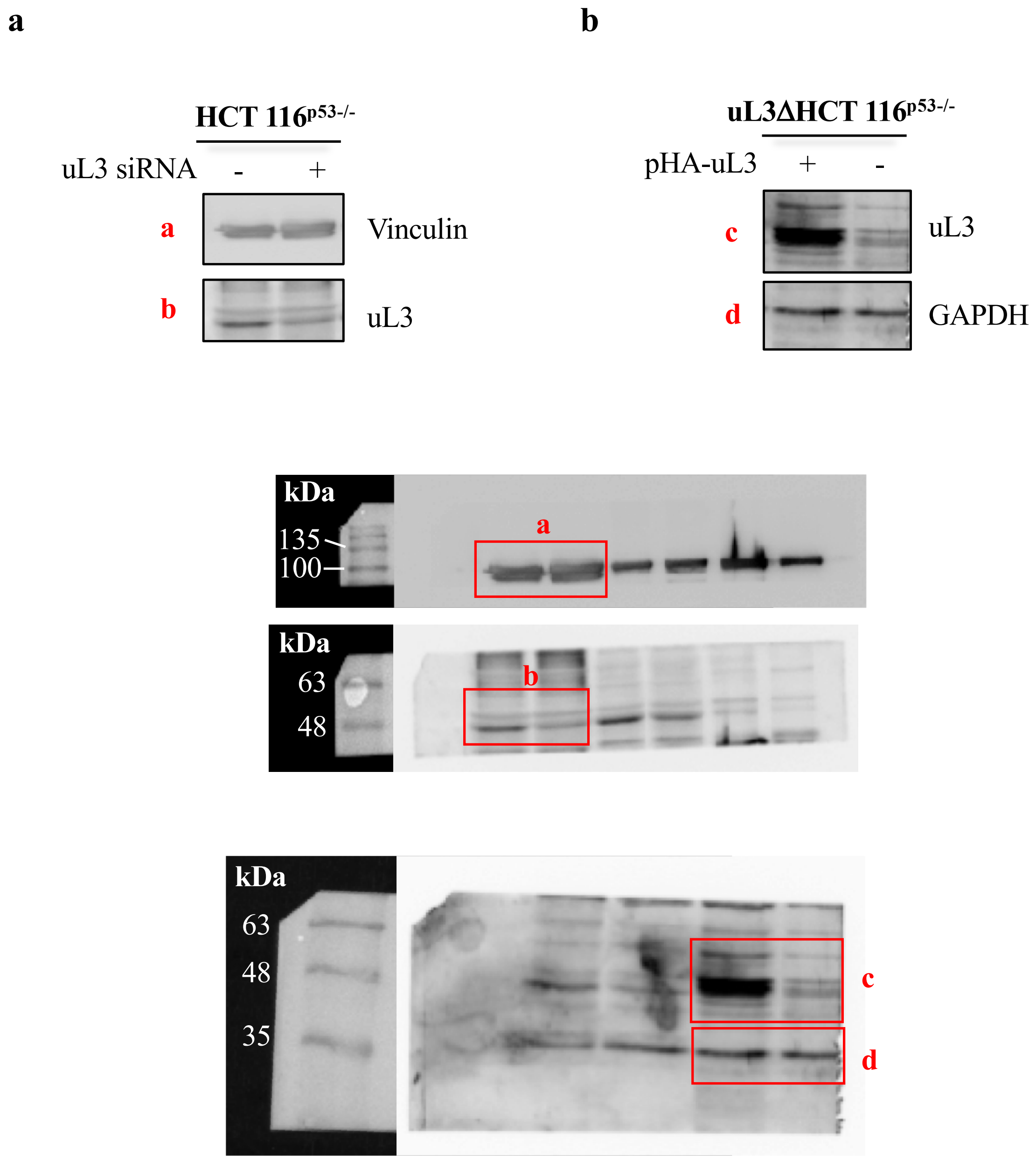

Supplementary Fig S13: full length blots of Fig S6
